# Risks of Skin, Hair, and Nail Supplements

**DOI:** 10.5826/dpc.1004a89

**Published:** 2020-10-26

**Authors:** Emily K. Burns, Ariadna Perez-Sanchez, Rajani Katta

**Affiliations:** 1 Baylor College of Medicine, Houston, TX, USA; 2 Department of Internal Medicine, University of Texas Health Science Center, San Antonio, TX, USA; 3 Department of Dermatology, McGovern Medical School at UT Health, Houston, TX, USA

**Keywords:** dietary supplements, nutrition, diet, toxicity, adverse effects

## Abstract

Skin, hair, and nail supplements, sometimes referred to as “beauty supplements” or “ingestible skin care,” are a large and growing industry. These products may contain vitamins and minerals, sometimes in very high doses. They may also contain herbs, hormones, microbes, or animal derivatives such as fish oils and collagen powders. Dietary supplements are regulated as foods, not as drugs, by the US Food and Drug Administration (FDA). Therefore, manufacturers do not need to provide any proof of safety, efficacy, or quality prior to sale. This is of serious concern, as many adverse effects due to supplement components have been reported.

The potential risks cover multiple categories. These include acute toxicities, such as choking, as well as chronic toxicities, such as increased risk of diabetes. Teratogenicity and interactions with drugs and laboratory testing have been documented in research studies. Other risks include potentially increased risk of cancer with long-term use, allergic reactions, and others. It is vital that physicians educate their patients on these risks. As no post-marketing surveillance programs are required for supplements, our understanding of supplement risks is incomplete. Physicians should be wary of these risks and encourage further research and regulation.

## Introduction

**Figure 1:**
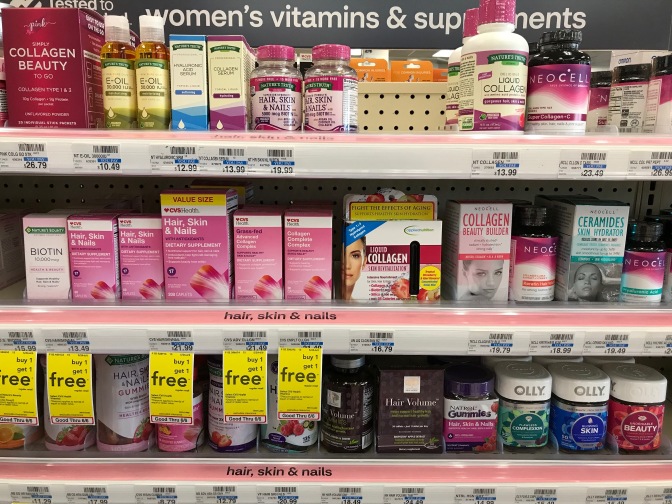
Multiple skin, hair, and nail supplements available for sale at a local retailer.

**Figure 2:**
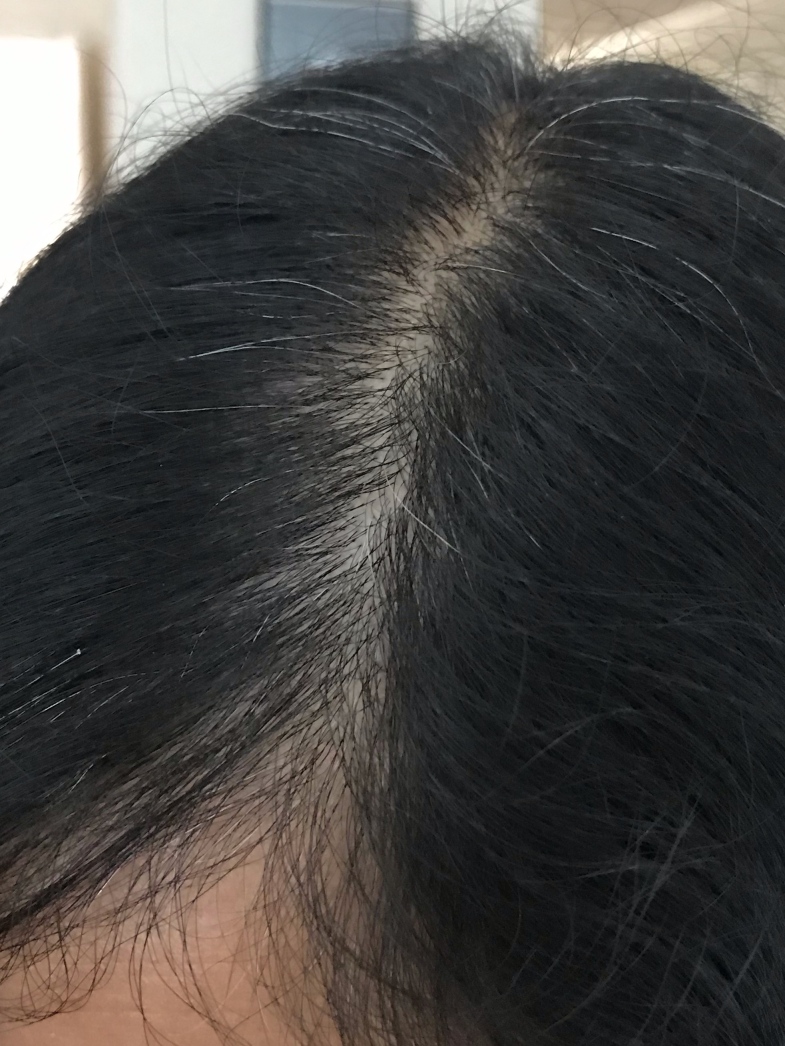
Patients with androgenetic alopecia, seen here with a widened part, may inquire about the use of skin, hair, and nail supplements.

**Figure 3:**
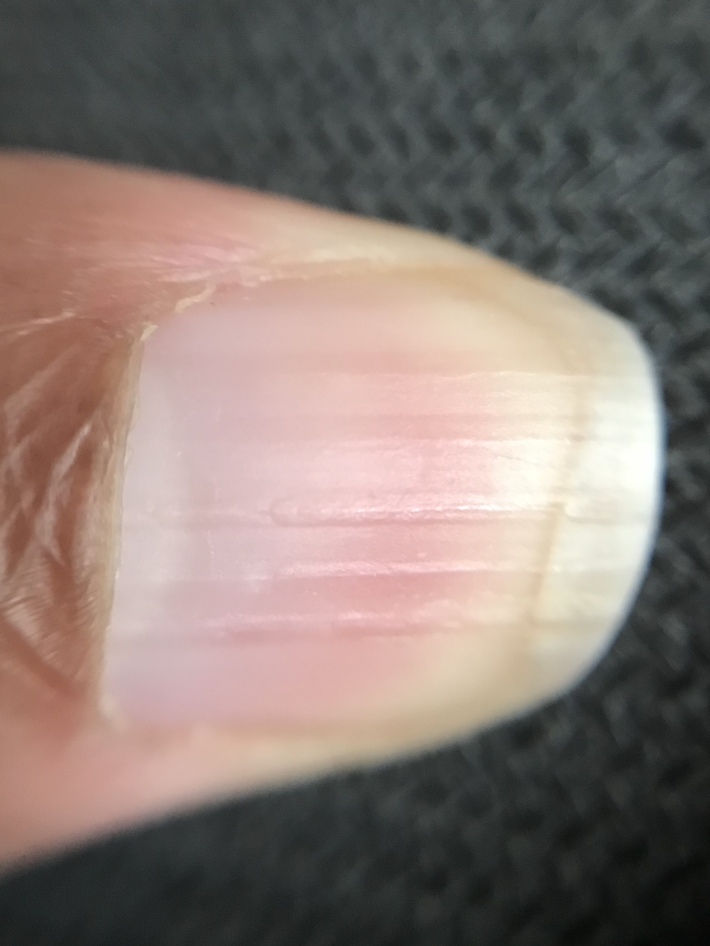
Patients with brittle nails may inquire about the use of nail supplements.

Skin, hair, and nail supplements have become a large and growing industry in the United States and worldwide. Sometimes referred to as “beauty supplements” or “ingestible skin care,” these dietary supplements continue to increase in popularity. They are frequently promoted on social media and by celebrities, sometimes as “natural” alternatives to cosmetic procedures. These products are sold online and in stores (Figure 1). Dietary supplements in general are widely used, with reports indicating that approximately half of US adults report using dietary supplements [1–4]. The global beauty supplement market was valued at $3.5 billion in 2016 and is projected to grow to $6.8 billion by the end of 2024, according to a report by Goldstein Research [5]. As more patients, such as those with androgenetic alopecia or brittle nails (Figures 2 and 3), express an interest in these supplements, it is imperative that dermatologists be able to answer their questions and provide guidance based on the medical literature. An overview of the potential risks is especially vital.

**Figure 4:**
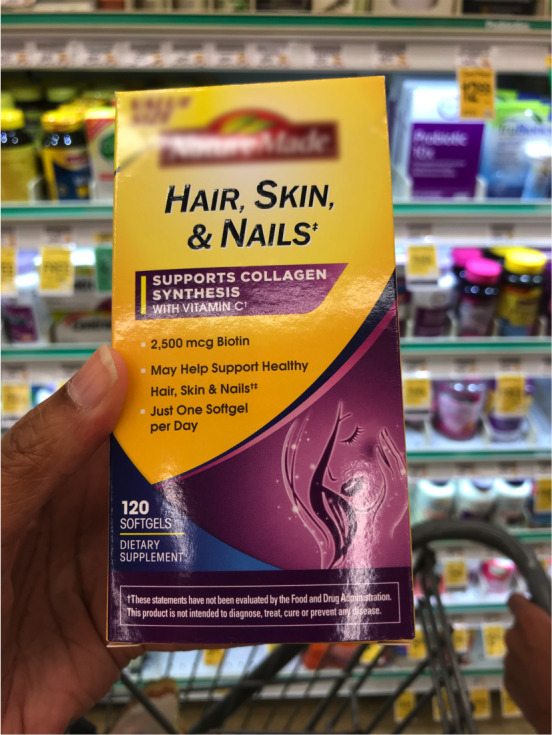
Example of a skin hair, and nail supplement, seen here with a health claim and a disclaimer.

**Figure 5:**
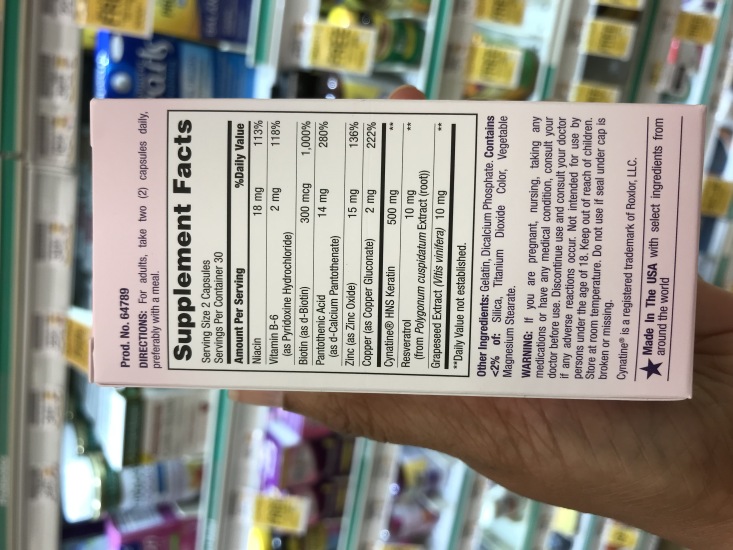
Example of the Supplement Facts label for a skin, hair, and nail supplement.

The category of skin, hair, and nail supplements includes many different ingredients, and educating patients on the potential risks of these ingredients is especially important. Vitamins are frequent ingredients, such as vitamins A, C, and multiple B vitamins, as well as minerals such as zinc and selenium. Some of these nutrients are included in supplements at very high doses (Figures 4 and 5). Herbal ingredients such as saw palmetto are common as well. Food components are included in this category, such as collagen powders and fish oils. Even hormones and microbes are marketed as having beneficial skin effects, including melatonin and probiotics [5].

The dietary supplement industry has minimal oversight from the US FDA. Supplements are regulated as foods, not as drugs, which has multiple implications in terms of safety and oversight [6,7]. By law, manufacturers of dietary supplements do not require approval by the FDA before launching a new product [8]. In essence, any company can bring to market a dietary supplement without having to first provide any evidence of efficacy or safety. Multiple vitamins, minerals, herbs, and other substances may be combined without having to first test for compatibility or interactions.

In addition, FDA requirements on warning labels are minimal [6]. Supplements with iron must warn about overdosing and poisoning in children [9]. If a manufacturer includes a structure/function claim on a supplement label, then they must also include a statement or “disclaimer.” This disclaimer indicates that the structure/function claim has not been evaluated by the FDA and that “this product is not intended to diagnose, treat, cure, or prevent any disease” [8]. Other warning requirements are lacking. There is no required warning label indicating risk of teratogenicity, even when demonstrated in research studies. The same applies to interactions with medications and laboratory tests, even when documented by research.

In this review, we describe several categories of potential risks posed by skin, hair, and nail supplements. In discussing these risks, physicians must emphasize to their patients the importance of multiple variables that impact how an individual may react to a particular supplement. These variables include age, the use of other medications, underlying medical conditions, differences in metabolism, and others.

We emphasize that there are a number of other risks not discussed here. Of special concern are those risks that may be severe but infrequent. Such risks may only be discovered in post-marketing surveillance programs. The supplement manufacturer is solely responsible and lacks supervision for ensuring pre-marketing safety and efficacy. Research and documentation of these parameters is completely optional. Similarly, post-marketing surveillance by the manufacturer is not required. Instead, it relies on reports from consumers, health care professionals, and industry members. In other words, the FDA does not actively monitor new dietary supplements, leading to a large gap in our knowledge of supplement safety.

## Acute Toxicities

A number of acute toxicities have been reported (Table 1). Reports indicate over 23,000 emergency department visits annually in the U.S. for supplement adverse events [10]. Among adults ≥65, pill-induced dysphagia or choking was associated with 37.6% of all emergency department visits for supplement adverse events, with micronutrients implicated in over 80% of cases [10]. The FDA recommends a pharmaceutical tablet size of less than 22 mm and requires reporting of tablet size on all new drug applications. By contrast, supplements do not have reporting requirements or size recommendations. Many products available in stores exceed 22 mm [10,11].

Melatonin, a hormone sometimes marketed for “beauty” sleep, carries a risk of sedation and may increase fall risk. In one trial, a single 3-mg dose resulted in impaired postural control in older adults [12].

In terms of physiologic effects, high doses of vitamin C have been associated with the formation of calcium oxalate kidney stones, particularly in patients with impaired renal function [13,14]. High doses have also been associated with acute hemolysis in those with G6PD deficiency [15]. High doses of B6 and B12 have been associated with the development of rosacea fulminans [16].

## Chronic Toxicities

Hair loss supplements may contain a number of different ingredients, including the minerals iron and selenium. Although supplemental iron has not shown benefit for hair loss in those with normal levels, iron is frequently found in dietary supplements advertised for hair loss [17]. The long-term use of iron in those who are not deficient, even at low doses, has resulted in symptoms of iron overload [18]. Common adverse effects of iron overdose are constipation, gastrointestinal upset, reduced zinc uptake, and iron overload in hemochromatosis [19].

Long-term selenium use is also concerning. Although the upper tolerable intake is 400 μg per day, concerns have been raised at 200 μg per day, a dose found in some hair loss supplements [20]. One randomized controlled trial (RCT) examined the effects of different doses of selenium as compared to a placebo in a country with moderately low selenium status at baseline. Researchers found that a 300 μg per day dose of selenium ingested daily for 5 years increased all-cause mortality as assessed 10 years later [21]. Epidemiological studies have raised concern as well. Multiple studies have noted that high plasma selenium levels may be associated with increased prevalence of type 2 diabetes, hyperglycemia, and dyslipidemia [22–24]. Selenium may also increase the incidence of type 2 diabetes. One RCT found that 200 μg per day in non-diabetic patients (average age of 63 years and average follow-up of 7.7 years) significantly increased risk for development of type 2 diabetes as compared to placebo [25]. Importantly, an exposure-response gradient was found across subgroups of plasma selenium levels [25]. In patients with preexisting type 2 diabetes, administration of selenium for 3 months resulted in statistically significant elevations in serum glycosylated hemoglobin A1c (HbA1c) levels, as well as fasting plasma glucose [26].

## Risk of Nutrient Overconsumption From Supplements With Dietary Sources

Another challenge with the use of dietary supplements is the parallel consumption of food (Table 2). Nutrients are derived from both supplements and foods, and this is a concern for certain nutrients. The US Food and Nutrition Board has published upper tolerable limits for 24 nutrients [27]. These nutrients should be maintained within an optimal level of intake: not too low, but also not too high. In the case of specific nutrients, certain foods may contain high levels already. Adding a supplement in these cases may quickly lead to consumption above the upper tolerable limits and result in multiple adverse effects [28]. For example, the recommended daily value (DV) for selenium in a healthy adult is 200 μg, and the upper tolerable intake level (UL) is 400 μg [27]. A single Brazil nut can contain up to 90 μg of selenium [29]. These values demonstrate the risk of a supplement containing the recommended DV in conjunction with just 3 Brazil nuts (270 μg). This combination exceeds the established UL, increasing the risk of adverse events.

Iron overload may occur even at low doses [18], raising concern about the risks of iron supplementation in conjunction with ingestion from high-iron foods.

## Drug Interactions

Supplements may interact with many prescription medications and laboratory tests. One literature review documented over 1,400 unique interactions with over 200 herbs and supplements [30]. Since no formal surveillance programs are required for supplements, it is imperative that physicians be alert for new reports.

In fact, despite a long history of biotin use in skin, hair, and nail supplements (Figures 4 and 5), it was only in 2017 that the FDA issued a warning about its potential for interactions with laboratory testing [31]. Impacted tests included those testing for thyroid and cardiac function. In a clinical trial, subjects were tested with specific biotinylated immunoassays before and after taking biotin 10 mg daily for 7 days. Biotin ingestion interfered with 9 out of 23 biotinylated immunoassays. Researchers found falsely decreased thyroid-stimulating hormone concentrations, raising the concern of misdiagnosing hyperthyroidism in a healthy individual, as well as falsely decreased NT-proBNP, a test used to help diagnose congestive heart failure [32]. Troponin levels, used to diagnose myocardial infarction, were also falsely decreased.

Another study evaluated the accuracy of urine pregnancy tests in women consuming biotin. In nonpregnant women who ingested biotin 10 mg daily for 7 days, urine samples were tested with a qualitative b-hCG urine pregnancy test. After day 3, 3 out of 4 tests revealed the absence of a control line. In other words, certain urine pregnancy tests may not function in women consuming biotin [33].

Reports in cardiology journals caution against the use of saw palmetto in patients taking warfarin due to the potential impacts on bleeding time. Case reports have described excessive intraoperative bleeding during a craniotomy [34], intraoperative floppy iris syndrome during a cataract procedure [35], and hematuria and coagulopathy in one patient [36]. The risk factors, frequency, and extent of this effect is not known, as one study in 10 volunteers found that saw palmetto ingestion did not affect platelet function tests in this small group [37].

## Teratogenicity and Effects on the Reproductive System

As current labeling laws in the United States do not require pregnancy category warnings on any supplements, physicians must counsel their patients of any potential teratogenicity risks. Saw palmetto (*Serenoa repens*) is one concerning supplement. This plant has a long history of use in Asia and among Native Americans, especially as a treatment for benign prostatic hypertrophy (BPH), and is frequently found in supplements advertised for that condition [38]. It inhibits 5-alpha-reductase, which prevents the conversion of testosterone to dihydrotestosterone [39,40]. Because of this ability, saw palmetto is also featured in a number of hair loss supplements advertised for hair loss in both men and women [20]. Saw palmetto, as a 5-alpha-reductase inhibitor, presents a severe teratogenicity risk. The administration of 5-alpha-reductase inhibitors to pregnant animals is associated with male offspring with abnormal male genitalia. Therefore, these drugs are labeled pregnancy category X, the category of greatest concern for pregnant women, by pharmaceutical companies [38]. Due to a lack of labeling regulations, no such warning is required for supplements, despite a similarly demonstrated mechanism of action.

High doses of vitamin A are also teratogenic, with risk particularly high before the seventh week of pregnancy, at a time when some women may not be aware of their pregnancy. Among pregnant women who averaged more than 10,000 IU per day of vitamin A orally (in the form of retinoid compounds), approximately 1 in 57 had a malformation owing to the supplement [42]. Similarly high doses may be found in some dietary supplements advertised for skin benefits, as in certain acne supplements [44].

For many nutrients, the risks related to ingestion of high doses are not known and have not been studied systemically. Zinc is often used in acne supplements and is one such nutrient that warrants further study. In one study, elevated levels of zinc in umbilical cord blood were associated with adverse neonatal neurobehavioral development [45].

Melatonin is another concerning supplement. Although sold as a dietary supplement, melatonin is actually a hormone that has important effects on circadian rhythms. Animal studies have indicated that melatonin has profound effects on reproductive organs, leading to concerns regarding its long-term use in a pediatric population [46].

## Allergies

Due to the lack of federal regulation, little is known about hypersensitivity reactions to supplements. Multiple supplement ingredients have been associated with both Stevens-Johnson syndrome and toxic epidermal necrolysis, including ascorbic acid and Chinese herbal supplements [47]. Diindolylmethane, sometimes used in acne supplements, has been associated with the severe systemic allergic reaction DRESS (drug reaction with eosinophilia and systemic symptoms) [48]. Cutaneous drug reactions and urticaria have been described in herbs used to treat skin conditions [49,50].

Additives used in many supplements, including dyes and preservatives, are well-known triggers of allergic reactions. Such reactions include urticarial reactions, fixed drug reactions, generalized dermatitis, and even anaphylaxis [50,51].

While the allergenicity of collagen powders derived from sources such as seafood is unknown, hydrolyzed fish collagen has been associated with anaphylaxis [52].

## Potential Cancer Risk

The role of micronutrients in chemoprevention and carcinogenesis is not completely understood. Animal research suggested that antioxidants, including beta-carotene, vitamin E, vitamin C and others, could have a chemoprotective effect. Observational studies found that individuals who consumed diets high in fruits and vegetables containing antioxidants were at lower risk of multiple cancers [53,54]. These promising results have not been observed when supplemental doses of antioxidants (as opposed to dietary doses) have been evaluated in large, population-based RCTs. In fact, in some cases supplementation may increase cancer risk.

Many supplements marketed as skin, hair, and nail supplements contain high levels of micronutrients. Some contain high levels of vitamins A, B6, B12, E, and selenium, all of which have been associated with higher cancer risk in various groups.

UV radiation enhances the formation of cutaneous free radicals, which play a pivotal role in the development of skin cancer (SC). Dietary antioxidants (AOs) are important in neutralizing free radicals, but dose (dietary as opposed to supplemental) and timing is critical [55]. Research on small animal models suggested that AO supplementation could prevent the development of SC [56]. However, in much of this research, AO supplementation occurred *before* exposure to UV radiation. In human studies, by contrast, AO supplementation often occurs after years of exposure to UV light [57].

In fact, supplementation with high doses of AOs may prove detrimental. In one study, researchers evaluated the effects of a combination supplement that contained promising micronutrients in the hope that it would reduce the risk of SC in women aged 35–60. This supplement contained vitamin C, vitamin E, beta-carotene, selenium, and zinc. Unfortunately, the incidence rate of SC in women consuming this supplement was significantly higher than those who took a placebo [57]. Another study examined the effects of selenium exposure. In this epidemiological study, the incidence of melanoma was 4 times higher in individuals exposed to high selenium levels from the environment than unexposed individuals [58].

This association between high-dose micronutrient supplementation and increased risk of cancer has been noted for other cancers as well, including lung cancer. Male smokers who took beta-carotene, a precursor to vitamin A, were at increased risk for lung cancer and cardiovascular disease in comparison to a placebo group. In fact, the trial was stopped early because the mortality rate was 17% higher in the treatment group [59,60]. Another study examined the effects of vitamins B6 and B12 supplementation and found a 30%–40% increased risk of lung cancer among male smokers [61].

In terms of other cancers, alpha-tocopherol (vitamin E) supplementation in healthy men increased the risk of prostate cancer by 17% [62]. Folic acid supplementation in healthy men also increased the risk of prostate cancer [63].

## Other Risks

A wide variety of other risks has been described from skin, hair, and nail supplements. Some include well-known risks, such as sedation from melatonin. Less well-known risks include hair loss that may result from high levels of selenium, vitamin A, and vitamin E [17]. Ironically, these are often found in supplements marketed for use in hair loss [64].

Other reported reactions are due, not to side effects from the active ingredients, but rather to the quality of the formulation. Quality concerns are a major concern with supplements, as the FDA does not require any proof of quality prior to sale. While manufacturers are required to follow current FDA Good Manufacturing Practices, the FDA is only able to inspect a small fraction of facilities every year. For the fiscal year 2019, 51% of dietary supplement manufacturing facilities in the US and abroad were cited for noncompliance with these practices [65]. Quality concerns are a serious issue, as multiple reports have described microbial contamination with bacteria [66] as well as with fungi [66,67]. Multiple studies have also reported adulteration with heavy metals, such as Ayurvedic medicines contaminated with lead, mercury, and arsenic [68], and collagen powders contaminated with cadmium [69]. Adulteration with prescription medications has also been described for multiple supplements [70].

In addition, labeling and manufacturing errors have led to serious side effects. A manufacturing error resulted in selenium supplements containing 200 times the labeled concentration, resulting in multiple cases of acute selenium toxicity [71], while a mislabeled and improperly formulated vitamin D supplement led to a patient consuming more than 1,000 times the recommended dosage [72].

## Conclusions

In accordance with FDA regulation, all prescription drugs must include a package insert detailing black box warnings, potential risks, and pregnancy category warnings. Supplements lack all of these warnings. In addition, no post-marketing surveillance programs are required. The results of these programs with prescription medications demonstrate their importance in detecting infrequent but severe side effects. Such programs have led to recalls of multiple promising prescription medications, such as the antihistamine terfenadine, linked to serious cardiac arrhythmias from drug interactions [73] and the anti-obesity drugs fenfluramine and dexfenfluramine, recalled due to reports of heart valve damage [74].

In discussing the risks of dietary supplements, we emphasize that our understanding of their safety and risk profiles is incomplete. Physicians should be wary of the ever-growing supplement industry and encourage further research and regulation.

## Figures and Tables

**Table 1:** Acute and Chronic Toxicities Due to Skin, Hair, and Nail Supplements (Selected Examples)

Acute Toxicity	Ingredient
Dysphagia or choking, particularly among adults ≥ 65 [10]	Tablet size >22 mm
Sedation and falling risk [12]	Melatonin
Calcium oxalate kidney stones [13,14] Acute hemolysis in patients with G6PD deficiency [15]	High-dose vitamin C
Rosacea fulminans [16]	High-dose vitamin B6 High-dose vitamin B12
**Chronic Toxicity**	
Constipation, gastrointestinal upset, reduced zinc uptake [18,19]	High-dose iron
Increased all-cause mortality [21] Increased incidence of type 2 diabetes, dyslipidemia, and hyperglycemia [22–25]	High-dose selenium

**Table 2:** Adverse Effects due to Skin, Hair, and Nail Supplements (Selected Examples)

Adverse Effect	Ingredient
**Interaction**	
Lab testing: thyroid-stimulating hormone, troponin, b-hCG and NT-proBNP tests [30–33]	Biotin (vitamin B7)
Warfarin (leading to risk of increased bleeding) [34–36]	Saw palmetto
**Teratogenicity**	
Ambiguous genitalia in male offspring [38]	Saw palmetto (5-alpha-reductase inhibitor)
Birth malformations [41–43]	High-dose vitamin A
**Allergic reactions**	
Anaphylaxis [52]	Hydrolyzed fish collagen
Drug reaction with eosinophilia and systemic symptoms [48]	Diindolylmethane (DIM)
Urticarial reactions, fixed drug reactions, generalized dermatitis, anaphylaxis [50,51]	Dyes and preservatives used in supplements
**Increased Cancer risk**	
Skin cancer in women [57]	Antioxidant supplement (vitamin C, vitamin E, beta-carotene, selenium, zinc)
Melanoma [58]	High-dose selenium
Lung cancer in smokers [60]	Beta-carotene
Lung cancer in smokers [61]	High-dose vitamins B6 and B12
